# Analytical Validation of a Novel MicroRNA Panel for Risk Stratification of Cognitive Impairment

**DOI:** 10.3390/diagnostics13132170

**Published:** 2023-06-26

**Authors:** Arzu Kunwar, Kenny Kwabena Ablordeppey, Alidad Mireskandari, Kira Sheinerman, Michael Kiefer, Samuil Umansky, Gyanendra Kumar

**Affiliations:** DiamiR Biosciences Laboratory, 2 Church Street South, Suite B05, New Haven, CT 06519, USA; akunwar@diamirbio.com (A.K.); kablordeppey@diamirbio.com (K.K.A.); amireskandari@diamirbio.com (A.M.); ksheinerman@diamirbio.com (K.S.); mkiefer@diamirbio.com (M.K.); sumansky@diamirbio.com (S.U.)

**Keywords:** analytical validation, mild cognitive impairment (MCI), Alzheimer’s disease (AD), neurodegenerative diseases, cognitive impairment, microRNA biomarkers, blood-based molecular diagnostics, expression profiling of circulating miRNA, LNA-based qPCR, TaqMan-based qPCR

## Abstract

We have been developing a novel approach to identify cognitive impairment-related biomarkers by profiling brain-enriched and inflammation-associated microRNA (miRNA) in plasma specimens of cognitively unimpaired and cognitively impaired patients. Here, we present an analytical validation of the novel miRNA panel, CogniMIR^®^, using two competing quantitative PCR technologies for the expression analysis of 24 target miRNAs. Total RNA from the plasma specimens was isolated using the MagMAX mirVana Kit, and RT-qPCR was performed using stem-loop-based TaqMan and LNA-based qPCR assays. Evaluation of RNA dilution series for our target 24 miRNAs, performed by two operators on two different days, demonstrated that all CogniMIR^®^ panel miRNAs can be reliably and consistently detected by both qPCR technologies, with sample input as low as 20 copies in a qPCR reaction. Intra-run and inter-run repeatability and reproducibility analyses using RNA specimens demonstrated that both operators generated repeatable and consistent Cts, with R^2^ values of 0.94 to 0.99 and 0.96 to 0.97, respectively. The study results clearly indicate the suitability of miRNA profiling of plasma specimens using either of the qPCR technologies. However, the LNA-based qPCR technology appears to be more operationally friendly and better suited for a CAP/CLIA-certified clinical laboratory.

## 1. Introduction

Due to increasing lifespans, especially in developed countries, the number of Alzheimer’s disease (AD) patients and individuals with a high risk of developing cognitive impairment is rapidly growing. It has been demonstrated that AD dementia is preceded by 10–20 years of disease development in the brain, initially without clinical symptoms (pre-symptomatic AD), and then is manifested as mild cognitive impairment (MCI), which progresses to dementia at a rate of 8–15% annually [1,2,3,4]. It is also known that not all MCI cases progress to AD, and some individuals diagnosed with cognitive decline return to cognitively unimpaired status [1]. Thus, there is an urgent need to identify biomarkers capable of identifying potential AD cases at their earliest stages, including MCI, among cognitively unimpaired individuals.

Existing cerebrospinal fluid (CSF) biomarker tests based on the analysis of proteins involved in AD development (beta amyloid, tau and p-tau, NfL) and positron emission tomography (PET) measures show strong diagnostic properties; however, their uses outside of very specialized clinics are limited due to invasiveness, cost, and restricted accessibility [5,6,7,8]. As a result, the utility of CSF and PET biomarkers in most primary and secondary care settings worldwide is limited, necessitating other diagnostic approaches for AD [9]. 

Screening for AD pathology with blood biomarkers is less invasive and likely less costly than CSF or neuroimaging markers, making it a more feasible option for use as a screening tool for primary care use, where most individuals will present with cognitive symptoms [8,10]. Although the development of blood biomarkers has been previously hindered by insufficient analytical sensitivities, recent studies suggest promising results using easily accessible and potentially scalable blood biomarker tests [7,10].

The usefulness of cell-free plasma microRNAs (miRNAs) enriched in different brain regions as diagnostic targets for AD pathology has been proposed by us [1,2,4,11,12] and other independent labs [13,14,15,16] in the past decade. The destruction of neurites and/or synapses in the pre-symptomatic phase of AD results in the release of brain-enriched miRNAs into the blood, potentially allowing the use of these targets as surrogates of synaptic loss from early events in AD development [4]. In addition, there are other biological processes, such as blebbing of the plasma membrane during late-stage apoptosis, exosome secretion, and other forms of active secretion, that result in the release of neurite/synapse miRNAs into the extracellular medium. These processes can also link the presence of brain-enriched targeted miRNAs with the early onset of brain damage that eventually results in AD pathology [1,2,4,12]. Indeed, we have identified plasma miRNA biomarker candidates for the risk of preclinical AD, MCI, and AD, as well as the risk of progression of pre-MCI to MCI and MCI to AD [1,2,4,17]. Our studies have also indicated that brain-enriched miRNAs may be useful targets for diagnostic assay development, not only for AD but also for other neurodegenerative diseases (NDs), such as frontotemporal dementia (FTD), Parkinson’s disease (PD), and amyotrophic lateral sclerosis (ALS) [18].

miRNAs are small, non-coding RNAs that control the post-transcriptional regulation of gene expression by binding to their target mRNAs and regulate degradation or repression of mRNA translation [3,19]. By controlling and regulating protein synthesis, miRNAs play an important role in cellular function and cell biology in both normal and dysregulated cells [3,9]. Expression profiling of circulating miRNAs in plasma and serum specimens can potentially be used for disease detection across a disease continuum and for the prediction of response to therapy [20,21,22]. Several methods are currently used to quantify miRNAs, including real-time quantitative PCR (qPCR), digital PCR, microarray, and high-throughput small RNA sequencing [23,24]. Among these, qPCR is the most common method used to detect low levels of miRNAs [23,24,25,26].

To understand the role of miRNA expression in cognitive impairment studies, plasma specimens from cohorts of cognitively unimpaired and various cognitively impaired study participants were secured for the isolation of RNAs. These were then tested for expression levels of 24 target miRNAs to determine the correlations between their levels and cognitive impairment [1,11,18]. We utilized a “miRNA ratio of expression” approach for data normalization and identification of miRNA pairs that might be relevant for differentiating cognitively unimpaired, age-matched controls (NC) from MCI and AD [1,11,27]. Initially, the ratio of expression analysis was carried out manually using the respective Ct values from the qPCR experiments [1,11]. Eventually, a proprietary custom software tool was developed to generate miRNA pair analysis data outcomes [1].

We created a novel 24-miRNA panel, CogniMIR^®^, consisting of miRNAs that are enriched in brain regions affected by AD. In this study, we performed an analytical validation of the CogniMIR^®^ panel by utilizing miRNA expression profiling with both stem-loop-based TaqMan and LNA-based qPCR technologies. Our results indicated that while both technologies were suitable for the miRNA expression analysis, Qiagen’s LNA-based qPCR technology was operationally better suited for large-scale testing of specimens in a CAP/CLIA-certified clinical laboratory.

## 2. Methods and Materials

### 2.1. Procurement of Plasma Specimens

The clinical plasma samples used in this study were collected at the Roskamp Institute, Sarasota, FL, USA, and IRB-approved written consent was obtained from each study participant recruited in the study. The samples were collected from 65–85-year-old, non-fasting study participants. Venous blood was collected in K2EDTA BD vacutainers (ref. No. 366643) and centrifuged at 4 °C for 15 min at 3000× *g*. The 0.5 mL plasma aliquots were transferred into Biosphere SC micro-tubes (S tubes; Sarstedt Cat no. 72.730.217) and frozen at −80 °C.

### 2.2. RNA Isolation from Plasma Specimens

RNA was isolated from clinical plasma samples using the MagMAX mirVana Total RNA Isolation Kit (Thermo Fisher Scientific, Waltham, MA, USA) as follows: 200 µL of plasma was digested with 10 µL of proteinase K in 90 µL of proteinase K buffer for 30 min at 65 °C, followed by the addition of 200 µL of lysis-binding mix and 40 µL of RNA-binding magnetic beads. A total of 540 µL of isopropanol was then added, and the samples were mixed and shaken for 15 min, followed by washing the RNA-bound beads with 300 µL of wash solution I and then with 300 µL of wash solution II on a magnetic stand. Subsequent to the treatment of the beads with 100 µL of Turbo DNase, 100 µL of rebinding buffer was added to facilitate RNA binding to the beads. A total of 200 µL of isopropanol was then added to precipitate the RNA. Beads with bound RNA were subsequently washed to remove contaminants, and the RNA was removed from the beads using 100 µL of elution buffer (provided with the kit) at 65 °C. Eluted RNA was stored at −70 °C. In general, 200 µL plasma samples yield 680 ng to 810 ng (6.8 ng/µL to 8.1 ng/µL) of RNA, with 260/280 ratio values around 1.58 to 1.61, as determined by Nanodrop. According to DiamiR’s standard protocol, prior to RT-PCR, 100 µL of eluted RNA was diluted with 225 µL of nuclease-free water to obtain a total of 325 µL of RNA, and this diluted RNA was used in all assays carried out in this study.

### 2.3. CogniMIR^®^ 24-miRNA Panel

The miRNA panel, shown below in Table 1, consists of 24 brain-enriched and inflammation-associated miRNAs, which were previously shown to be effective at differentiating between cognitive impairment and a healthy cognitive state [1,4,11,12,18].

### 2.4. Preparation of Synthetic Positive miRNA Controls for the Expression Analysis

Synthetic RNA oligos for the 24 miRNAs in our panel were synthesized at Integrated DNA Technologies (Coralville, IA, USA) and used as positive controls for the assay. Our quantification marker mix was prepared with 10 ng/µL PolyA in 10 mM Tris pH 7.0, which contained equal amounts of the 24 miRNA oligos. A total of 1 µL (6.02 × 10^13^ copies) of the 100 µM stock solution for each of the 24 miRNA oligos was mixed with 76 µL of nuclease-free water to generate a 1 µM working solution. This was followed by further dilutions to achieve a 6.25 nM working solution (3.75 × 10^9^ copies/µL) containing each of the 24 miRNAs. From this, a 12 pM working solution (1 × 10^7^ copies/µL) was prepared using 10 ng/µL Poly A in 10 mM Tris pH 7.0 as a diluent. The 1:10 serial dilutions were further prepared from the 12 pM solution to achieve 200,000 to 20 copies per 2 µL of a qPCR reaction.

### 2.5. Determination of miRNA Expression Using Stem-Loop-Based TaqMan RT-qPCR Technology

Isolated RNA was reverse transcribed into cDNA by using the TaqMan MicroRNA Reverse Transcription Kit. A total of 24 individual RT master mixes were prepared. An amount of 11 µL of this master mix contained the following volumes of reagents: 5.16 µL of nuclease-free water, 0.15 µL of 100 mM dNTPs, 1.5 µL of 10X RT buffer, 0.19 µL of RNase inhibitor (20 U/µL), 1 µL of MultiScribe reverse transcriptase enzyme, and 3 µL of 5X primer. For the RT reaction, 11 µL of the miRNA-specific master mix was added to 4 µL of RNA sample in a 96-well plate. The 96-well plate map was designed to test 12 miRNAs, and thus, we used two 96-well plates to assay the 24 targeted miRNAs. The RT reaction was carried out on a thermal cycler for 30 min at 16 °C and at 42 °C for an additional 30 min. The reaction was terminated at 85 °C for 5 min, followed by holding the reaction at 4 °C.

The 24 cDNA products were amplified in separate wells using the TaqMan miRNA qPCR assay. A total of 6 µL of qPCR master mix (containing 5 µL of TaqMan universal master mix II (no UNG), 0.5 µL of nuclease-free water, and 0.5 µL of 20X TaqMan probe) was added to 4 µL of 1:1 diluted RT reaction product. The 384-well plate map was designed such that 12 miRNAs were tested in triplicate in 6 plasma RNA samples, along with an NTC well and 1 quantification marker. The 384-well plate was run on a Quantstudio 7 Flex programmed as follows: 1 cycle at 95 °C for 10 min (enzyme activation), followed by 40 cycles at 95 °C for 15 s (denaturation) and 60 °C for 60 s (annealing and extension). The raw Cts of all triplicate assays were then exported from the Quantstudio for the data analysis.

### 2.6. Determination of miRNA Expression Using LNA-Based Qiagen RT-qPCR Technology

Prior to the RT step, a large batch of synthetic spike-in (UniSp6) was prepared as per the miRCURY LNA RNA Spike-in Kit instructions. For the reverse transcription step, 20 µL of RT master mix (containing 6 µL of nuclease-free water, 8 µL of 5X miRCURY RT reaction buffer, 4 µL of 10X miRCURY RT enzyme mix, and 2 µL of synthetic RNA spike-in) was added to 20 µL of isolated RNA plated in predetermined wells of a 96-well plate. The RT step was carried out for 60 min at 42 °C, followed by inactivation of the reaction at 95 °C for 5 min, and then placed at 4 °C. Since the procedure allowed for the sequence-independent RT of all the miRNAs, each well contained the cDNA for all the targeted miRNAs.

The cDNA product was amplified using Qiagen’s Locked Nucleic Acid (LNA) RT-qPCR technology for the 24 targeted miRNAs in the panel. Sample-specific, primer-deficient master mixes were prepared in such a way that 10 µL of this master mix would contain 3.7 µL of nuclease-free water, 0.20 µL of ROX reference dye, 5 µL of 2X miRCURY LNA SYBR Green Master Mix, and 1.07 µL of RT reaction product. A total of 10 µL of sample-specific master mix was added to the appropriate wells of the miRCURY LNA miRNA Custom PCR Panel of a 384-well plate. The custom PCR panel was designed such that 12 samples were tested for 32 targets (24 targeted miRNAs, along with additional miRNAs and controls suggested by Qiagen). qPCR was carried out on a Quantstudio 7 Flex programmed with initial heat activation at 95 °C for 2 min (1 cycle), followed by denaturation at 95 °C for 10 s, and annealing/extending at 60 °C for 56 s (40 cycles). Melt-curve analysis was set at 60–95 °C. The raw Cts of all assays were exported for data analysis.

### 2.7. Determination of Limit of Detection for the 24 miRNAs Using TaqMan and LNA-Based Qiagen qPCR Technologies

A dilution series of pooled synthetic RNA oligos (quantification marker mix) representing the 24 target miRNAs (from 200,000 to 20 copies of total input in a qPCR assay) was used to determine the lower limit of detection of both the TaqMan and Qiagen technologies. The goal of this study was to determine the minimum copy number of the 24 miRNAs that could reliably and reproducibly be detected with Ct values in the <36–40 range for all of the 24 miRNAs. Expression of the 24 miRNAs in the quantification marker mix dilution series was evaluated on 2 different days using both the TaqMan and Qiagen technologies. The performances of all the miRNA assays were assessed by generating standard curves and calculating the slope and R^2^ values.

### 2.8. Determination of Intra-Run and Inter-Run Reproducibility Using TaqMan and LNA-Based Qiagen qPCR Technologies

To determine the repeatability and reproducibility of Ct values for all 24 miRNAs in the CogniMIR^®^ panel, the intra-run and inter-run correlations between the Cts were assessed. In this study, 4 samples (3 clinical plasma specimens and 1 quantification marker mix of 24 synthetic miRNAs) were tested in duplicate by 2 operators on 2 different days using both the TaqMan and Qiagen technologies. The intra-run Ct correlations were identified between the replicates obtained from both days for each operator by creating a scatterplot graph, while the inter-run Ct correlations were identified between Cts obtained from two different days from each operator.

### 2.9. Statistical Analysis

The Excel software 2010 program was used for routine qPCR data analysis. The R software 4.2.1 programming language (RStudio 2022.02.3 Build 492) was used to analyze the quality control parameters (UniSp3 monitors PCR efficiency, and UniSp6 monitors RT efficiency) of the Qiagen platform and the limit of detection analysis for both technologies. The raw Ct data of the UniSp3, UniSp6, and positive control dilution series were used for this analysis by utilizing the ggplot2 package.

## 3. Results and Discussion

### 3.1. CogniMIR^®^ 24-miRNA Panel

We established a panel of 24 brain-enriched and inflammation-associated miRNAs detectable in plasma as a highly promising epigenetic biomarker of MCI and AD [1,4,11,18]. We initially selected miRNA biomarker candidates among those enriched in specific brain regions affected by the disease, such as the hippocampus in early MCI due to AD, present in synapses, and detectable in blood plasma. Since AD development is often accompanied by neuroinflammation, inflammation-associated miRNAs were also included in our miRNA panel [18]. The levels of the 24 miRNAs showed promise in our earlier studies as biomarkers of synaptic loss and neurodegeneration [1,2,4,17,18,27]. Analysis of miRNAs enriched in affected brain regions and a combination of neurite/synapse miRNAs with neuronal body miRNAs and inflammation-associated miRNAs are informative for cognitive impairment-related risk stratification. The 24 miRNAs comprising the panel are shown in Table 1.

### 3.2. RT-qPCR Performance Evaluation of TaqMan and Qiagen’s LNA Technology for miRNA Quantification

To evaluate the reverse transcription and qPCR performance of TaqMan versus Qiagen’s LNA platform, we quantified the expression (Ct values) of 24 synthetic miRNAs representing the CogniMIR^®^ panel (quantification marker mix) in a dilution series ranging from 200,000 copies to 20 copies. The qPCR reactions were performed on two different days and by two operators. Standard curves were generated and analyzed for all 24 miRNAs using both technologies, as shown in Table 2. The slope values, PCR efficiency, and R^2^ values were found to be within the acceptable range, which indicates that all 24 synthetic RNAs were efficiently and consistently measured in the synthetic oligo dilution series by both technologies [28].

### 3.3. Determination of Limit of Detection Using Qiagen’s LNA and TaqMan’s Stem-Loop Technologies

**Figure 1:**
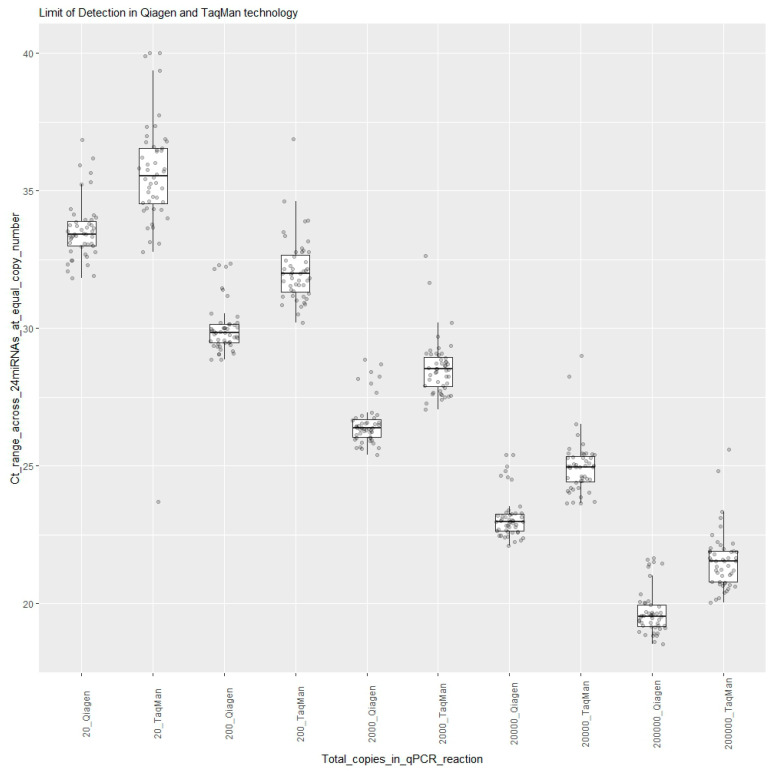
Limit of detection using Qiagen’s LNA and TaqMan’s stem-loop technologies.

The limit of detection was determined by measuring the expressions of 24 synthetic RNAs in the dilution series (quantification marker mix), ranging from 200,000 copies to 20 copies, in a qPCR reaction tested on 2 days using both the TaqMan and Qiagen technologies. Dilution series lower than 20 copies were not tested to avoid stochastic variation in amplification and subsampling error. This resulted in measuring Ct values for each of the 24 miRNAs for each day for each copy number. Boxplots were created using R programming, where each boxplot represented a synthetic RNA oligo dilution, with 48 Ct data values shown as points in Figure 1. Figure 1 shows that the ranges of Cts for 2000 copies for all 24 miRNAs were 26 Ct to 28 Ct in Qiagen and 27 Ct to 32 Ct in TaqMan. For 200 copies, the ranges were 28 Ct to 32 Ct in Qiagen and 30 Ct to 37 Ct in TaqMan. For the lowest input of 20 copies in a qPCR reaction, the ranges were 32 Ct to 37 Ct in Qiagen and 32 Ct to 40 Ct in TaqMan. Although the Ct values from the Qiagen technology were lower than those from the TaqMan technology, all 24 target miRNAs of the panel could be detected in the sample input with as low as 20 copies in qPCR reactions using both technologies. 

### 3.4. Determination of Intra-Run and Inter-Run Reproducibility and Repeatability of TaqMan Assays for 24 miRNAs

To determine the repeatability and reproducibility of the detection of all 24 miRNAs of the panel in the TaqMan assay platform, the intra-run and inter-run correlations between the Cts were assessed in 4 samples (3 clinical plasma samples and 1 quantification marker mix). In both the intra-run and inter-run studies, these four samples were tested for the twenty-four miRNAs in duplicate by two operators on Day 1 and Day 2.

**Figure 2:**
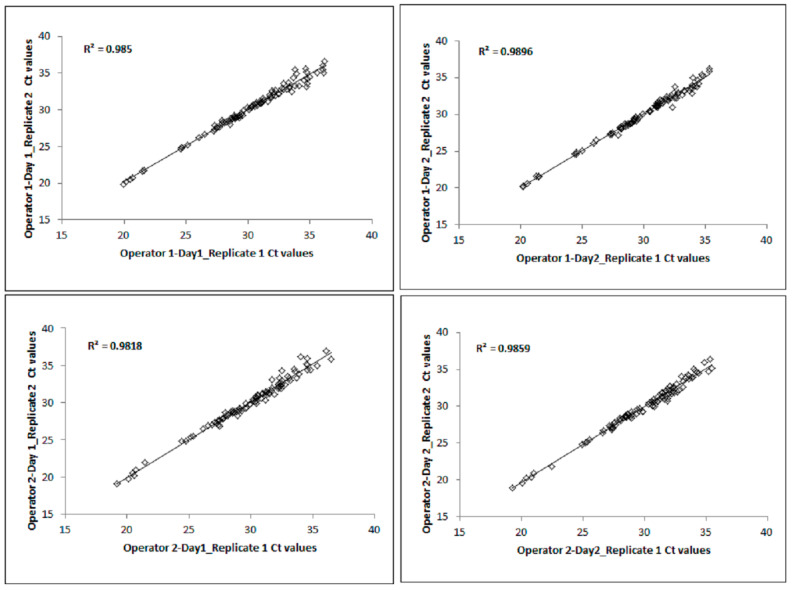
Intra-run Ct correlations of 24 miRNAs using the TaqMan technology.

The intra-run Ct correlation between the replicates obtained from both days for each operator was determined by creating a scatterplot and identifying the R^2^ value, as shown in Figure 2. For the intra-run analysis, each graph consisted of *n* = 192 Ct data points (4 samples × 24 miRNAs × 2 replicates). For each operator, the Cts from the first replicates were plotted against the Cts from the second replicates for Day 1 and Day 2. The R^2^ value between Replicate 1 and Replicate 2 on Day 1 and Day 2 for Operator 1 was 0.98. Similarly, the R^2^ value between Replicate 1 and Replicate 2 on Day 1 and Day 2 for Operator 2 was also 0.98. The intra-run Ct correlation between the two replicates for both operators on the two days was R^2^ = 0.98.

**Figure 3:**
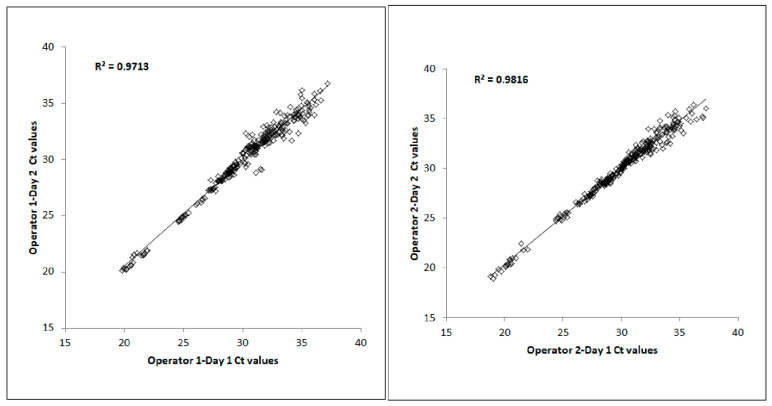
Inter-run Ct correlations of 24 miRNAs using the TaqMan technology.

The inter-run Ct correlation between Day 1 and Day 2 for each operator was determined by creating a scatterplot and identifying the R^2^ value, as shown in Figure 3. For the inter-run analysis, each graph consisted of *n* = 384 Ct data points (4 samples × 24 miRNAs × 2 replicates × 2 days). The inter-run Ct correlations between the two days for Operator 1 and Operator 2 were R^2^ = 0.97 and 0.98, respectively. For each operator, the Cts from both replicates from Day 1 were plotted against the Cts from both replicates from Day 2. The R^2^ value between Day 1 and Day 2 for Operator 1 was 0.97. Similarly, the R^2^ value between Day 1 and Day 2 for Operator 2 was 0.98.

### 3.5. Determination of Intra-Run and Inter-Run Reproducibility and Repeatability of Qiagen Assays for 24 miRNAs

To determine the repeatability and reproducibility of the expression of all 24 miRNAs in the Qiagen platform, the intra-run and inter-run correlations between the Cts were assessed in four samples (three clinical plasma samples and one quantification marker mix). In both the intra-run and inter-run studies, these four samples were tested for the twenty-four miRNAs in duplicate by two operators on Day 1 and Day 2.

**Figure 4:**
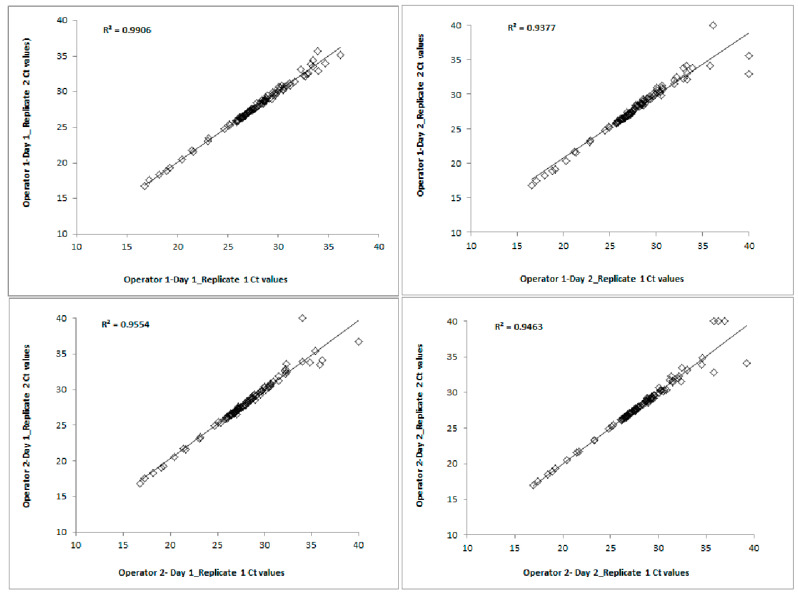
Intra-run Ct correlations of 24 miRNAs using the Qiagen technology.

The intra-run Ct correlation between the replicates obtained from both days for each operator was determined by creating a scatterplot and identifying the R^2^ value, as shown in Figure 4. For the intra-run analysis, each graph consisted of *n* = 192 Ct data points (4 samples × 24 miRNAs × 2 replicates). For each operator, the Cts from the first replicates were plotted against the Cts from the second replicates for Day 1 and Day 2. The R^2^ values between Replicate 1 and Replicate 2 on Day 1 and Day 2 for Operator 1 were 0.99 and 0.94, respectively. Similarly, the R^2^ values between Replicate 1 and Replicate 2 on Day 1 and Day 2 for Operator 2 were both 0.95. The intra-run Ct correlation between the two replicates for both operators on both days was R^2^ = 0.93 to 0.99. 

**Figure 5:**
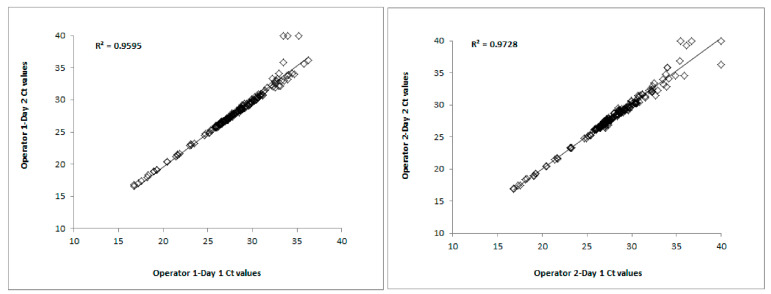
Inter-run Ct correlations of 24 miRNAs using the Qiagen technology.

The inter-run Ct correlation between Day 1 and Day 2 for each operator was determined by creating a scatterplot and identifying the R^2^ value, as shown in Figure 5. For the inter-run analysis, each graph consisted of *n* = 384 Ct data points (4 samples × 24 miRNAs × 2 replicates × 2 days). For each operator, the Cts from both replicates from Day 1 were plotted against the Cts from both replicates from Day 2. The R^2^ value between Day 1 and Day 2 for Operator 1 was 0.96. Similarly, the R^2^ value between Day 1 and Day 2 for Operator 2 was 0.97. The inter-run Ct correlations between the two days for Operator 1 and Operator 2 were R^2^ = 0.95 and 0.97, respectively.

### 3.6. Quality Control Analysis for Reverse Transcription and qPCR Assay for the Qiagen Technology

To determine the reverse transcription and qPCR assay performance among the custom Qiagen qPCR plates, we used UniSp6 and UniSp3 controls, respectively. These controls were designed to quantify reverse transcription and PCR amplification efficiency and act as positive controls for these steps.

**Figure 6:**
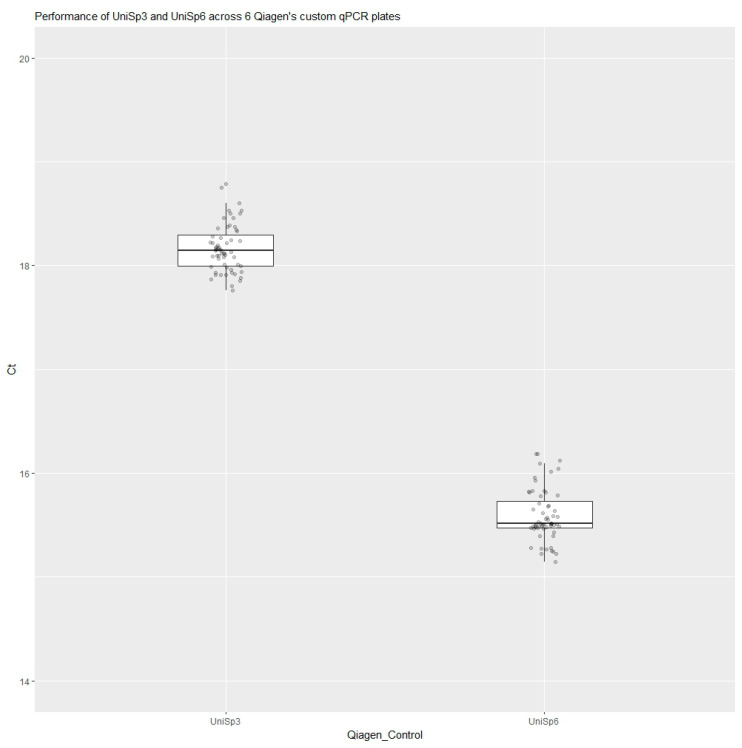
Quality control analysis of six of Qiagen’s custom qPCR plates.

UniSp6 is a synthetic RNA spike-in template, a large batch of which was prepared before the study. A DNA template and corresponding DNA primers for UniSp3 were pre-plated in Qiagen’s custom qPCR plates. As part of our quality control analysis, 6 samples (5 quantification marker dilutions and 1 NTC) were tested in 2 of Qiagen’s custom qPCR plates, and 4 samples (3 plasma samples and 1 quantification marker) were tested in 4 of Qiagen’s custom qPCR plates, totaling 60 Ct data points each for UniSp3 and UniSp6. Data obtained from the six Qiagen custom qPCR plates were used to generate the boxplots shown in Figure 6. The Ct values for UniSp6 ranged from 15 Ct to ∼16 Ct, while the Ct values for UniSp3 ranged from ∼18 Ct to 19 Ct. UniSp6 and UniSp3 can also be used as inter-plate calibrators. As designed, a fluctuation in the detection trends of these controls serves as an indicator of errors in the assay set-up, instrument failure, or the presence of inhibitors in the samples from the RNA isolation step. A stable detection of UniSp6 (cDNA synthesis control) in the Ct range of 15 to 16 across all samples tested indicates that the analytical design of the plates meets the required quality control criteria. In this study, we observed stable Cts within the samples and across the qPCR plates, indicating efficient reverse transcription. Since the UniSp3 was pre-aliquoted in the wells of the custom qPCR plates, the variations in UniSp3 within a plate and across plates were theoretically minimal. Stable detection of UniSp3 (qPCR performance control) in the Ct range of 18 to 19 across all samples tested indicated that the analytical performance of these plates met the quality control criteria.

### 3.7. Advantages/Disadvantages of TaqMan vs. Qiagen Technologies for Routine Clinical Testing in a CAP/CLIA Environment 

We evaluated both the TaqMan and Qiagen LNA qPCR technologies for routine miRNA expression profiling of clinical plasma specimens using the CogniMIR^®^ miRNA panel in a clinical laboratory under CAP/CLIA requirements. While TaqMan utilized an individual RT for each target miRNA, a stem-loop design for primers, and a sequence-specific fluorescent probe for the detection, the Qiagen technology utilized universal reverse transcription of all the miRNAs, followed by qPCR with LNA-based primers and SYBR green for the fluorescent detection.

The data generated in this study indicated that both the TaqMan and Qiagen platforms resulted in reproducible and repeatable data. The TaqMan platform required independent RT reactions that were carried out for each of the 24 miRNA assays, while Qiagen required a single universal RT reaction for all 24 target miRNAs, thus simplifying the workflow and significantly saving time. A single operator could test 90 RNA samples in >4 weeks using the TaqMan protocol, while the Qiagen protocol would allow a person to test these 90 RNA samples in 1 week, making this platform faster and operationally more efficient.

For the TaqMan platform, two qPCR plates are required to test twenty-four targeted miRNAs for a single clinical sample, which would eventually introduce variability between these plates. However, only one qPCR plate is required to test all twenty-four targeted miRNAs using the Qiagen technology, eliminating the possibility of inter-plate variation. The universal RT reaction in the Qiagen technology is also expected to reduce technical variation. Utilization of pre-plated reagents for the Qiagen LNA technology is also expected to minimize experimental variability by minimizing variances due to primer preparation and pipetting. Furthermore, Qiagen’s custom qPCR plates have pre-aliquoted qPCR performance controls, such as UniSp3 and spike-in controls, such as UniSp6, to monitor cDNA synthesis and UniSp2, UniSp4, and UniSp5 to monitor RNA isolation. The presence of these internal controls in each custom qPCR plate aids in tracking quality metrics of the data generated in each batch and helps to troubleshoot the failed experiments. This also allows for continuous monitoring of the batch-to-batch variability over the course of time, which is important for any CAP/CLIA-certified clinical laboratory.

Another advantage of Qiagen’s LNA technology RT-qPCR procedure is the hot-start procedure, which allows for the set-up of the PCR reaction at room temperature without the risk of primer-dimer formation. Furthermore, the SYBR green PCR mix in Qiagen’s LNA technology contains an inert blue dye that helps to visually monitor the uniformity of the qPCR mix in the wells. Furthermore, the flexibility in designing a custom plate with the Qiagen technology will allow us to optimize the study in the future, if needed. These factors all combine to add additional layers of controls and quality metrics in day-to-day lab operations.

## 4. Conclusions

Blood biomarkers have shown their utility by being easily accessible and cost-effective targets for identifying and diagnosing several NDs, such as AD and MCI. We identified 24 miRNA biomarker candidates enriched in brain regions affected by ND pathologies to assess the risks of preclinical AD, MCI, and AD, as well as other NDs. This novel 24-miRNA panel, CogniMIR^®^, showed differentiation between cognitively unimpaired vs. MCI vs. AD in previously reported studies [1,11,18,27].

One of the challenges of blood biomarker testing is creating a scalable and reproducible assay that can be reliably performed in a high-volume laboratory setting with low turnaround time (TAT). In this study, we performed an analytical validation of the CogniMIR^®^ panel by carrying out miRNA expression profiling using two different qPCR technology platforms, namely the stem-loop-based TaqMan and Qiagen LNA-based qPCR technologies.

The presence of inhibitors in clinical samples may interfere with and reduce the efficiency of either the RT or the qPCR steps, resulting in assay failures. One distinct advantage of the Qiagen LNA platform is the inclusion of spike-in internal controls for both RT efficiency (UniSP6) and PCR efficiency (UniSP3). Failure of the PCR reaction can arise from the presence of inhibitors in clinical specimens or issues with the primers, the master mix, or plates. Since clinical samples and the UniSp3 control are plated in separate wells, failure of UniSp3 will be indicative of primer/master mix/plate issues. The inclusion of these positive controls allows a clinical lab to quickly and efficiently determine reasons behind QNS (quality not sufficient) signals, such as RT failure vs. qPCR failure, and implement corrective actions.

The results of our study indicate the suitability of miRNA profiling of plasma specimens using either of the qPCR technologies. Both platforms resulted in reliable and reproducible results, with strong R^2^ values for the quantification of miRNA in clinical samples. However, the LNA-based qPCR technology, which utilizes a single universal RT reaction and contains internal controls for both the RT and qPCR steps, appeared to be operationally friendlier than TaqMan and better suited for automation and large-scale testing of specimens in a CAP/CLIA-certified clinical laboratory.

## Figures and Tables

**Table 1:** CogniMIR^®^ 24 miRNA panel.

Number	miRNAs	Enrichment
1	Let-7e-5p	Cerebellum, Midbrain, Pituitary Gland
2	miR-107	Frontal Cortex, Pituitary Gland, Hippocampus, Midbrain
3	miR-125b-5p	Hippocampus, Frontal Cortex, Cerebellum, Pituitary Gland
4	miR-127-3p	Pituitary Gland, Midbrain, Frontal Cortex
5	miR-128-3p	Hippocampus, Frontal Cortex, Hypothalamus
6	miR-132-3p	Pituitary Gland, Hippocampus
7	miR-134-5p	Midbrain, Hippocampus, Pituitary Gland
8	miR-146a-5p	Inflammation associated
9	miR-155-5p	Inflammation associated
10	miR-16-5p	Ubiquitous/Pituitary Gland
11	miR-181a-5p	Midbrain, Frontal Cortex, Inflammation associated
12	miR-323-3p	Frontal Cortex, Hippocampus, Midbrain
13	miR-335-5p	Pituitary Gland, Hippocampus
14	miR-370-3p	Frontal Cortex, Pituitary Gland
15	miR-382-5p	Pituitary Gland, Hippocampus, Frontal Cortex
16	miR-409-3p	Pituitary Gland
17	miR-433-3p	Pituitary Gland, Midbrain
18	miR-451a	Ubiquitous/Pituitary Gland, Midbrain, Frontal Cortex
19	miR-487b-3p	Pituitary Gland, Midbrain
20	miR-491-5p	Frontal Cortex, Hippocampus, Pituitary Gland
21	miR-7-5p	Pituitary Gland, Frontal Cortex, Hippocampus
22	miR-874-3p	Hippocampus, Cerebellum
23	miR-9-5p	Frontal Cortex, Midbrain, Hippocampus, Cerebellum
24	miR-99b-5p	Midbrain, Pituitary Gland, Frontal Cortex, Cerebellum, Hippocampus

**Table 2:** The RT−qPCR performance of TaqMan and Qiagen’s LNA technology reagents for the 24 miRNAs in the CogniMIR^®^ panel.

Comparison of Average Slope, Average R2 and Average PCR Efficiency Values Generated from Standard Curves between TaqMan and Qiagen Technologies						
miRNA Targets	Average Slope_Qiagen	Average Slope_TaqMan	Average R2_Qiagen	Average R2_TaqMan	Average PCR Efficiency_Qiagen (%)	Average PCR Efficiency_TaqMan (%)
let-7e-5p	−3.44	−3.45	1.000	0.999	95.15	95.02
miR-107	−3.41	−3.48	1.000	0.999	96.66	93.72
miR-125b-5p	−3.59	−3.52	0.997	0.767	89.89	92.29
miR-127-3p	−3.31	−3.51	1.000	1.000	100.38	92.68
miR-128-3p	−3.41	−3.41	0.997	0.998	96.90	96.53
miR-132-3p	−3.57	−3.59	0.999	1.000	90.50	90.53
miR-134-5p	−3.36	−3.56	0.999	0.999	98.43	90.94
miR-146a-5p	−3.72	−3.70	0.993	0.997	85.57	87.02
miR-155-5p	−3.67	−3.45	0.997	0.999	87.46	94.99
miR-16-5p	−3.37	−3.62	0.996	0.997	98.30	89.06
miR-181a-5p	−3.47	−3.60	1.000	0.997	94.02	89.54
miR-323a-3p	−3.48	−3.53	1.000	0.998	93.70	92.71
miR-335-5p	−3.55	−3.56	0.999	1.000	91.42	90.98
miR-370-3p	−3.35	−4.18	0.999	0.993	99.13	73.39
miR-382-5p	−3.49	−3.35	1.000	0.996	93.33	99.10
miR-409-3p	−3.46	−3.50	0.999	1.000	94.88	93.06
miR-433-3p	−3.33	−3.53	1.000	0.999	99.73	92.48
miR-451a	−3.56	−3.54	1.000	0.999	90.94	92.47
miR-487b-3p	−3.44	−3.62	0.998	0.997	95.39	88.89
miR-491-5p	−3.37	−3.61	0.998	0.999	98.40	89.40
miR-7-5p	−3.48	−3.14	1.000	0.994	93.72	110.52
miR-874-3p	−3.33	−3.52	0.998	1.000	99.92	92.51
miR-9-5p	−3.42	−3.64	0.998	0.999	96.58	88.65
miR-99b-5p	−3.41	−3.57	0.999	0.999	96.56	90.70

## Data Availability

The data presented in this study are available upon request from the corresponding author. The data are not publicly available due to proprietary considerations.
